# Enhancing neural operator learning with invariants to simultaneously learn various physical mechanisms

**DOI:** 10.1093/nsr/nwae198

**Published:** 2024-06-06

**Authors:** Siran Li, Chong Liu, Hao Ni

**Affiliations:** School of Mathematical Sciences, Shanghai Jiao Tong University, China; Institute of Mathematical Sciences, ShanghaiTech University, China; Department of Mathematics, University College London, UK

## Abstract

We discuss the recent advancement in PDE learning, focusing on Physics Invariant Attention Neural Operator (PIANO). PIANO is a novel neural operator learning framework for deciphering and integrating physical knowledge from PDEs sampled from multi- physical scenarios.

Partial differential equations (PDEs) play a fundamental role in the modelling and analysis of a wide range of physical and geometric problems. Numerous numerical techniques, classical and new, have been proposed to approximate PDE solutions, aiming at attaining high accuracy and efficiency. Most recently, by utilising deep neural networks to represent PDE solutions, machine learning (ML) methods have emerged as a revolutionary tool that demonstrates enormous potential to overcome the curse of dimensionality and to deal with complex geometries.

The *neural operator* approach, which uses neural networks to learn the differential operator, stands out as arguably the most promising ML approach to simulate physical systems [1,4]. It nonetheless faces two major challenges: (i) it is only applicable to PDEs generated by a single physical mechanism and (ii) it requires access to high-level physical information governing the PDEs, which is infeasible or expensive to collect in real-world applications.

To this end, Zhang *et al.* [3] proposed a novel neural operator learning framework—the *physics invariant attention neural operator* (PIANO)—for deciphering and integrating physical knowledge from PDEs sampled from *multi-physical* scenarios. Such PDEs, most notably, contain various physical invariants (PIs), e.g. PDE coefficients and boundary/initial data.

In contrast to abundant literature focusing on one single fixed PDE (e.g. [4,5]), Zhang *et al.* [3] tackled the dataset generated by a family of PDEs with varying parameters arising from multi-physical processes. PIANO (Fig. 1) is proposed to enhance the neural operator method by incorporating the inherent invariance of PDEs.

**Figure 1.**
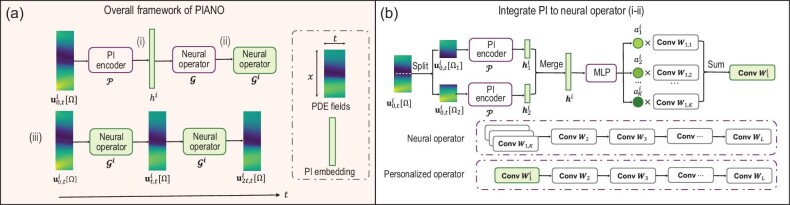
Flowchart of PIANO for learning the PDEs sampled from multi-physical scenarios. Left: flowchart of the overall framework of PIANO. Right: illustration of the ‘split-and-merge’ trick and the network architecture, which effectively integrates the PI embedding to the neural operator. Adapted from [3].

To illustrate the idea, consider the following schematic initial-boundary value problem of PDEs:


(1) \begin{eqnarray*}
\left\lbrace\begin{array}{lll}
&\partial _t u = \mathcal {R}(u, \theta _{\mathcal {R}}),\\
&u(x, 0) = u_0(x), \\
&\mathcal {B}(u)[x,t, \theta _{\mathcal {B}}]=0,\\ &\qquad\qquad\qquad (x, t) \in \Omega \times [0,T].
\end{array}\right.\!\!\!\!\!
\end{eqnarray*}

Here $\mathcal {R}$ and $\mathcal {B}$ are the differential and boundary operators, respectively, governed by global parameters $\theta =(\theta _{\mathcal {R}}, \theta _{\mathcal {B}})$. Zhang *et al.* [3] focus on the case in which $\theta$ is independent of *t*.

The learning task of PIANO is to predict the next *t*-frame solution given the past *t*-frame solution, using a dataset consisting of solutions governed by diverse parameters. Compared with [2], an essential feature of the work of Zhang *et al.* [3] is that it assumes *no prior knowledge* about the PDE parameters.

PIANO consists of two branches: (i) a PI encoder to extract invariant representation and (ii) a personalised operator to predict the complementary field representation of each PDE. Contrastive learning is employed to learn the embedding of each PDE in a data-driven fashion. In addition, three physics-aware cropping strategies are proposed to enhance consistency with the inherent invariance of the PDE system. Extensive numerical results on Burgers’ equation and the Navier–Stokes equations show that the proposed PIANO consistently outperforms the existing methods by reducing the relative errors by 13.6%–82.2%. Moreover, the supervised learning tasks of predicting the PDE coefficients demonstrate the usefulness of the learned PI representation in downstream tasks.

In conclusion, PIANO proposed by Zhang *et al.* [3] provides a novel, general framework for learning PDEs arising from multi-physical processes. It significantly improves empirical performance, is applicable to general backbone models and further inspires the design of neural operators for foundation models of PDE learning. Future research could explore the theoretical underpinnings of PIANO and extend its numerical validation to higher-dimensional problems. It is also of great interest to extend PIANO to the out-of-distribution generalisation tasks and transfer learning, noting that Zhang *et al.* [3] only studied such possibilities of the PI encoder. Moreover, the idea of utilising personalised encoders derived from larger models—which enables PIANO to accelerate the inference time while preserving the expressive power—opens up new avenues for the design of foundational models for PDE learning and the study of complex multi-physical problems in science and engineering.
